# Romidepsin inhibits Ras-dependent growth transformation of NIH 3T3 fibroblasts and RIE-1 epithelial cells independently of Ras signaling inhibition

**DOI:** 10.1186/1750-2187-4-5

**Published:** 2009-08-16

**Authors:** Ariella B Hanker, Kevin D Healy, Jean Nichols, Channing J Der

**Affiliations:** 1 Curriculum in Genetics and Molecular Biology, University of North Carolina at Chapel Hill, Chapel Hill, NC, USA; 2 Lineberger Comprehensive Cancer Center, University of North Carolina at Chapel Hill, Chapel Hill, NC, USA; 3 Gloucester Pharmaceuticals, Cambridge, MA, USA; 4 Department of Pharmacology, University of North Carolina at Chapel Hill, Chapel Hill, NC, USA

## Abstract

**Background:**

Despite intensive effort, currently no effective anti-Ras therapies have successfully reached clinical application. Previous studies suggest that the histone deacetylatse (HDAC) inhibitor romidepsin, which is currently in clinical trials for the treatment of multiple malignancies, can block Ras-dependent signaling and growth transformation. These studies suggest that mutational activation of Ras may be a useful biomarker for sensitivity to romidepsin and that the anti-tumor activity of this HDAC inhibitor may involve inhibition of Ras effector-mediated signaling.

**Results:**

To rigorously assess romidepsin as an antagonist of Ras, we utilized two well-characterized cell models for Ras transformation. We found that romidepsin blocked the anchorage-dependent and -independent growth of NIH 3T3 fibroblasts and RIE-1 epithelial cells transformed by all three Ras isoforms. However, romidepsin treatment also blocked growth transformation caused by other oncoproteins (B-Raf and ErbB2/Neu), suggesting that romidepsin is not selective for Ras. We also observed striking differences in romidepsin-mediated growth inhibition between transformed NIH 3T3 fibroblasts compared to RIE-1 epithelial cells, suggesting that the mechanism by which romidepsin blocks transformation is dependent on cellular context. Finally, we found that romidepsin did not inhibit Ras activation of the ERK and AKT effector pathways in NIH 3T3 and RIE-1 cells, suggesting that romidepsin does not directly antagonize Ras.

**Conclusion:**

Taken together, our results suggest that romidepsin is not selective for Ras-transformed cells and that the anti-tumor activity of romidepsin is not due to direct inhibition of Ras function.

## Background

Romidepsin (also called FK228, depsipeptide) is a bicyclic depsipeptide that was isolated from *Chromobacterium violaceum *and potently inhibits tumor growth [1,2]. The histone deacetylase (HDAC) family of enzymes was identified as the biologic target of romidepsin [3]. Romidepsin is a potent inhibitor of class I HDACs and, to a lesser extent, inhibits class II HDACs [4]. HDACs are involved in chromatin remodeling and transcriptional silencing, including the epigenetic silencing of several tumor suppressors [5]. Altered HDAC activity has been found in several cancers [5,6] and HDAC inhibitors have been shown to reverse cancer-associated epigenetic changes and cause growth arrest, differentiation, and apoptosis in cancer cell lines [6]. Therefore, romidepsin and other HDAC inhibitors have emerged as promising therapeutics for the treatment of cancer [4,6-8]. Presently, romidepsin and other HDAC inhibitors are under Phase I and II clinical evaluation [9].

The mechanistic basis for the anti-tumor activity of HDAC inhibitors remains poorly understood and is a topic of intense investigation [4,6-8]. While altered gene transcription is believed to be an important component of HDAC inhibitor activity, there is also evidence for altered function of signal transduction regulators. For example, there is evidence that mutational activation of H-Ras increases sensitivity to romidepsin-induced apoptosis and growth inhibition and that this HDAC inhibitor may block the function and signaling of the H-Ras oncoprotein [10,11]. The three *RAS *oncogenes (*HRAS*, *KRAS *and *NRAS*) are mutationally activated in ~30% of all cancers and are implicated in promoting multiple aspects of the malignant cancer phenotype [12-15]. Therefore, considerable effort has been made to develop anti-Ras inhibitors for cancer treatment. However, selective and clinically active Ras inhibitors have not yet been identified [13]. Romidepsin was described originally as an agent that reverted the transformed morphology of H-Ras-transformed NIH 3T3 mouse fibroblasts [1,16]. Romidepsin was further shown to inhibit the proliferation of H-Ras-transformed NIH 3T3 and C3H10T1/2 mouse fibroblasts [1,17]. However, *HRAS *mutations are rarely seen in human cancers (4%), and whether romidepsin also inhibits transformation caused by the Ras isoforms most commonly mutated in human cancers (*KRAS *and *NRAS*; 21 and 8%, respectively [18]) has not yet been addressed. This issue is important in light of growing evidence for isoform differences in Ras function [19]. Furthermore, while there is limited evidence that Ras-transformed cells display enhanced sensitivity to romidepsin-induced apoptosis [10,11], whether *RAS *mutation status will be a useful molecular determinant for HDAC inhibitor sensitivity is not yet known.

Ras functions as a GTP/GDP regulated switch that relays cellular signals involved in cell proliferation, differentiation, and survival [20]. In its GTP-bound state, Ras binds and activates a variety of effectors, including the Raf serine/threonine kinases and the phosphatidylinositol 3-kinases (PI3Ks), resulting in phosphorylation and activation of ERK and AKT, respectively. Several studies observed that romidepsin treatment inhibited Ras activation of ERK and AKT [17,21-23], although the relevance of these activities to romidepsin anti-tumor activity has not been established. Furthermore, other studies have made conflicting observations regarding the effects of romidepsin on Ras signaling [10,24].

To directly address the ability of romidepsin to antagonize Ras-mediated growth transformation and signaling, we utilized two model cell systems where mutationally activated Ras alone is sufficient and necessary for growth transformation. Ras-transformed NIH 3T3 and RIE-1 cells have been used extensively to study Ras-mediated transformation and signaling [25-27]. Our data suggest that romidepsin can inhibit growth transformation caused by mutant Ras, but is not selective for Ras-transformed cells. In contrast to previous observations that found that oncogenic Ras increased romidepsin-induced caspase-3 cleavage [10,11,17,28], we did not find that Ras rendered cells more sensitive to romidepsin-induced caspase-3 or PARP cleavage at concentrations that blocked growth transformation. Instead, our results suggest that romidepsin causes cell cycle arrest in these cell lines. Finally, our results reveal that romidepsin treatment did not inhibit ERK and AKT activity, suggesting that romidepsin does not directly antagonize Ras function.

## Methods

### Cell culture and generation of stable cell lines

NIH 3T3 mouse fibroblasts were maintained in Dulbecco's modified Eagle's medium (DMEM) supplemented with 10% calf serum (Colorado Serum Company, Denver, CO). RIE-1 rat intestinal epithelial cells were maintained in DMEM supplemented with 10% fetal calf serum (Sigma-Aldrich, St. Louis, MO). To generate mass populations of NIH 3T3 and RIE-1 cell lines stably expressing ectopic oncogenic proteins, infectious retrovirus of the pBabe-puro vectors encoding mutationally activated human H-Ras (G12V), K-Ras4B (G12V), N-Ras (G12D), B-Raf (V600E), and rat ErbB2/Neu (NeuT; V664E) was generated and used to stably infect NIH 3T3 and RIE-1 cells. Stable cell lines were maintained in growth medium supplemented with 1 μg/mL puromycin (Sigma-Aldrich).

### Proliferation assays

Stably transformed cells were trypsinized and 1000 cells/well (NIH 3T3) or 500 cells/well (RIE-1) were seeded in replicates of 8 in 96-well plates. The next day, medium was replaced with growth medium supplemented with DMSO, or 1, 3, or 5 nM romidepsin. Viable cells were quantified 72 h post-treatment with an MTT (3-(4,5-dimethyl-2-thiazolyl)-2,5-diphenyl-2H-tetrazolium bromide; Sigma) assay as described previously [29].

### Growth transformation assays

Mass populations of NIH 3T3 and RIE-1 cells stably infected with the empty pBabe-puro vector or encoding oncogenic proteins were analyzed in soft agar assays as we have described previously [30,31]. In triplicate, 2 × 10^4 ^NIH 3T3 or 10^4 ^RIE-1 cells were suspended in growth medium containing 0.4% agar and 0 (dimethylsufoxide; DMSO; vehicle only), 1 or 3 nM romidepsin. Media containing 0, 1, or 3 nM romidepsin was replenished weekly. Cultures were maintained at 37°C for two to three weeks, at which point viable colonies were stained with 500 μL of 0.2 mg/mL MTT viability stain (dissolved in PBS) and incubated at 37°C for a minimum of 1 h [29]. The number of viable colonies per plate was quantified using ImageJ software at a threshold of 80.

### Morphological reversion assays

NIH 3T3 and RIE-1 stable cell lines were seeded at a density of 3 × 10^4 ^and 4 × 10^4 ^cells per well, respectively, in 6-well plates. The next day, cells were treated in duplicate with 0.1% DMSO (vehicle control), 5 nM romidepsin, or 10 μM U0126 (Promega, Madison, WI). Following 48 h of treatment, morphologic changes were monitored by visual inspection using phase-contrast microscopy and representative fields were photographed using the 10× objective of an inverted phase-contrast microscope (Nikon).

### Immunoblotting and antibodies

Cells growing in log phase were treated with 0.1% DMSO, 5 nM romidepsin, 10 μM U0126, or 10 μM LY294002 (Promega) for 24 h. Alternatively, cells were treated with either 5 nM romidepsin for 72 h, or with 1 μM STS for 24 h as a positive control for induction of apoptosis. Cells were rinsed twice in ice-cold 1× PBS and then lysed in buffer containing 1% NP40, 1% deoxycholic acid, 0.1% SDS, 150 mM NaCl, and 50 mM Tris-HCl (pH 7.4), supplemented with EDTA-free protease inhibitor cocktail (Roche Applied Science) and phosphatase inhibitor cocktail I and II (Sigma-Aldrich). Lysates were normalized for protein concentration [determined with the Bio-Rad protein assay (Hercules, CA)], mixed with an equal volume of 2× protein sample buffer, resolved by SDS-PAGE, and transferred onto Immobilon P membranes (Millipore, Bedford, MA). The following primary antibodies were used for western blot analysis: anti-caspase-3, anti-PARP, anti-p42/44 MAPK (ERK1 and ERK2), anti-phospho-T202/Y204-p42/44 MAPK (ERK1 and ERK2), anti-AKT, anti-phospho-S473-AKT, anti-phospho-S608-Rb (Cell Signaling Technology, Danvers, MA); anti-B-Raf (Santa Cruz Biotechnology, Santa Cruz, CA); anti-pan-Ras, anti-p21^CIP1/WAF1 ^(CalBiochem); anti-cyclin D1 (NeoMarkers, Fremont, CA); anti-β-actin, and anti-vinculin (Sigma-Aldrich).

## Results

### Romidepsin inhibits proliferation of transformed NIH 3T3 and RIE-1 cells, but does not exhibit enhanced sensitivity for Ras-transformed cells

**Figure 1:**
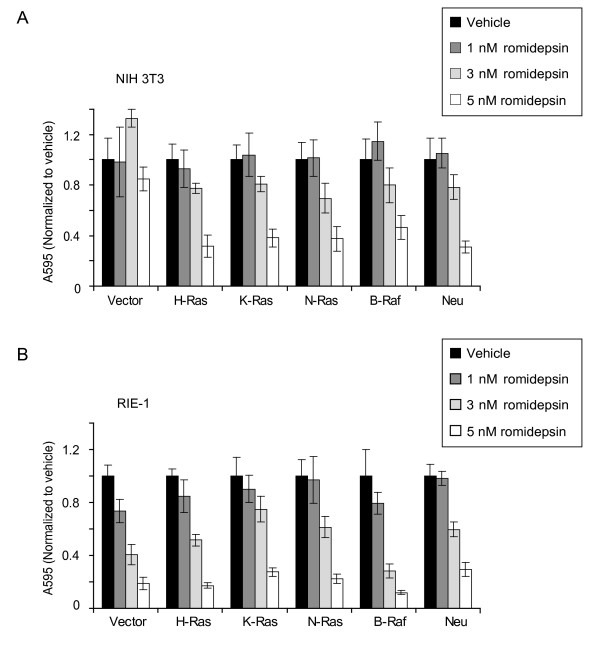
**Romidepsin inhibits the anchorage-dependent growth of Ras-transformed NIH 3T3 and RIE-1 cells**. NIH 3T3 cells (A) and RIE-1 cells (B) stably-infected with the empty pBabe-puro vector or encoding constitutively active human H-Ras(G12V), K-Ras(G12V), N-Ras(G12D), or B-Raf(V600E), or rat ErbB2/Neu(V664E) were seeded on day 0. On day 1, cells were treated with complete growth medium supplemented with DMSO (Vehicle), or 1, 3, or 5 nM romidepsin. After 72 h of romidepin treatment (day 3), cell viability was measured using the MTT assay. Data shown are the average ± SD of eight replicate wells and are representative of three independent experiments.

To rigorously evaluate the possible mechanism of romidepsin anti-tumor activity, we stably transformed NIH 3T3 mouse fibroblasts and RIE-1 epithelial cells with the oncogenic forms of H-, K-, and N-Ras, as well as with oncogenic B-Raf and ErbB2/Neu. To determine if romidepsin can selectively inhibit the growth of Ras-transformed cells, the anchorage-dependent proliferation of these cells was quantified following 72 h treatment with romidepsin. Treatment with 1 nM romidepsin did not drastically inhibit the anchorage-dependent proliferation of H-, K-, and N-Ras-transformed cells, but significant inhibition was seen at 3 to 5 nM. In contrast, treatment with up to 5 nM caused no significant impact on the growth of nontransformed NIH 3T3 cells (Figure 1A). These data extend previous studies which showed that H-Ras-transformed fibroblasts were more sensitive to romidepsin than nontransformed cells [17,24]. Similarly, 3 to 5 nM romidepsin also inhibited the growth of ErbB2/Neu-transformed cells. This result is consistent with the fact that ErbB2/Neu transformation of NIH 3T3 cells is dependent on Ras activation [32]. B-Raf(600E)-transformed NIH 3T3 cells also exhibited sensitivity to romidepsin growth inhibition at 3 or 5 nM. Since Raf transformation is Ras-independent, this result suggests that romidepsin inhibition of Ras-induced proliferation does not involve direct inhibition of Ras.

To extend our analyses to an epithelial cell type, Ras-transformed RIE-1 cells were also generated and tested for anchorage-dependent proliferative capacity in the presence of romidepsin. Like transformed NIH 3T3 cells, all oncogene-transformed RIE-1 cells showed significant inhibition at 3 nM. Interestingly, unlike the NIH 3T3 cells, transformed RIE-1 cells did not display an enhanced sensitivity to romidepsin (Figure 1B). Instead, we observed that the proliferation of nontransformed and transformed RIE-1 cells showed similar sensitivity to romidepsin treatment. Therefore, our data suggest cell context differences in romidepsin activity, where oncogenic Ras or Raf selectively sensitize NIH 3T3 but not RIE-1 cells to romidepsin.

### Romidepsin disrupts cell cycle regulators but does not promote caspase-3 cleavage in NIH 3T3 and RIE-1 cells

**Figure 2:**
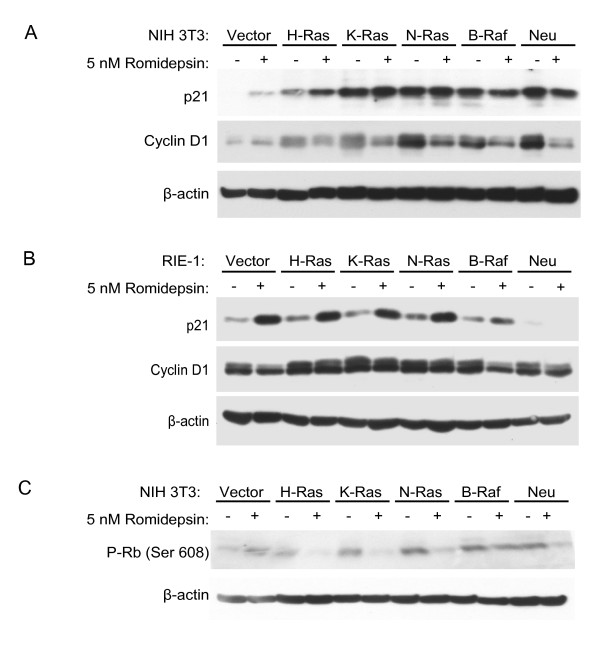
**Romidepsin affects expression of cell cycle regulators**. NIH 3T3 (A, C) or RIE-1 (B, D) cells stably expressing the indicated proteins were treated with DMSO (Vehicle) or the indicated concentration of romidepsin for 24 h. Cell lysates were analyzed by western blotting with antibodies to cyclin D1, p21, phospho-S608-Rb, and β-actin (loading control). Data shown are representative of at least two independent experiments.

Similar to our results above, previous studies found that romidepsin inhibited the growth of nontransformed and Ras-transformed cells [17]. However, these studies found that romidepsin inhibition of nontransformed C3H10T1/2 mouse fibroblasts was due to cell cycle arrest, whereas inhibition of Ras-transformed C3H10T1/2 cells was due to induction of apoptosis [17]. These studies suggested that Ras activation rendered cells sensitive to romidepsin-induced apoptotic activity. In order to determine whether the growth inhibition we observed by romidepsin was due to increased apoptosis or cell cycle arrest, we used western blot analysis to examine the expression levels of the cyclin-dependent kinase (CDK) inhibitor p21^CIP1 ^in response to romidepsin treatment in transformed and nontransformed NIH 3T3 and RIE-1 cells. As we have shown previously, p21 expression was increased in Ras-transformed NIH 3T3 and RIE-1 cells (Figure 2) [33]. Romidepsin slightly increased p21 expression in nontransformed as well as H-Ras-transformed NIH 3T3 cells (Figure 2A). Romidepsin did not upregulate p21 expression in other transformed cells. In contrast, romidepsin treatment strongly induced p21 expression in vector control and all Ras-transformed RIE-1 cells (Figure 2B). We also determined if romidepsin modulates expression of the cell cycle regulator cyclin D1. As we showed previously, cyclin D1 levels were increased in the Ras-transformed NIH 3T3 and RIE-1 cell lines (Figure(s) 2A and 2B) [33]. Treatment with romidepsin partially decreased cyclin D1 expression in transformed NIH 3T3 cells, but not in the nontransformed vector controls (Figure 2A). In contrast, romidepsin did not strongly affect cyclin D1 expression in the RIE-1 cells (Figure 2B). These results suggest that romidepsin may inhibit proliferation, in part, by upregulating p21 or downregulating cyclin D1 and inhibiting cell cycle progression.

Cyclin D1 and p21 function upstream of the retinoblastoma tumor suppressor protein (Rb). Thus, we asked whether romidepsin affected Rb phosphorylation levels, indicative of Rb inactivation. Romidepsin significantly reduced Rb phosphorylation in Ras-transformed NIH 3T3 cells, but not in nontransformed cells (Figure 2C). This reduction in Rb phosphorylation correlates with the sensitivity to romidepsin observed in the cell viability assays (Figure 1A): romidepsin blocked the proliferation of Ras-transformed cells more strongly than nontransformed NIH 3T3 cells. However, we failed to observe a significant decrease in Rb phosphorylation in RIE-1 cells treated with romidepsin (data not shown). Taken together, these data indicate that romidepsin blocks cell cycle progression of transformed NIH 3T3 and RIE-1 cells.

**Figure 3:**
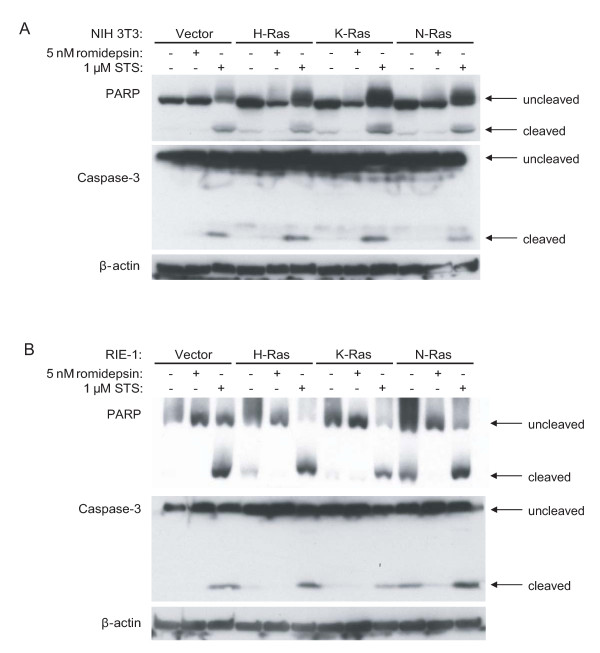
**Romidepsin does not induce cleavage of caspase-3 or PARP**. NIH 3T3 cells (A) and RIE-1 cells (B) stably infected with pBabe.puro vector or encoding H-Ras(G12V), K-Ras(G12V), or N-Ras(G12D) were treated with 5 nM romidepsin for 72 h or 1 μM staurosporine (STS) for 24 h. Cell lysates were analyzed by western blotting with antibodies to PARP, caspase-3, and β-actin (loading control). Data shown are representative of at least three independent experiments.

Previous studies have shown that romidepsin preferentially promoted apoptosis and caspase-3 cleavage in mutant Ras-transformed cells [10,11,17,24]. We also asked whether romidepsin promotes cleavage of caspase-3 in our stable cell lines, using the same conditions and romidepsin concentrations that we used in the cell viability assays. As a positive control to induce apoptosis, we treated cells with staurosporine (STS). As expected, STS treatment caused cleavage of caspase-3 and its target PARP. However, we did not observe enhanced cleavage of caspase-3 or PARP following 72 h of romidepsin treatment of Ras-transformed NIH 3T3 or RIE-1 cells (Figure 3). Interestingly, we observed elevated caspase-3 and PARP cleavage in untreated N-Ras-transformed RIE-1 cells, suggesting that these cells undergo apoptosis at a higher rate than other Ras-transformed or untransformed cells (Figure 3B). These observations are consistent with the lack of obvious morphologic alterations indicative of apoptosis in romidepsin-treated cells, but seen in STS-treated cells (data not shown). We also failed to observe annexin V staining in romidepsin-treated cells (data not shown). Thus, in contrast to previous observations [10,11,17,24], we did not find that Ras-transformed cells showed enhanced sensitivity to romidepsin-induced apoptosis under the conditions in which romidepsin inhibited cell proliferation.

### Romidepsin inhibits the anchorage-independent growth of transformed NIH 3T3 and RIE-1 cells

**Figure 4:**
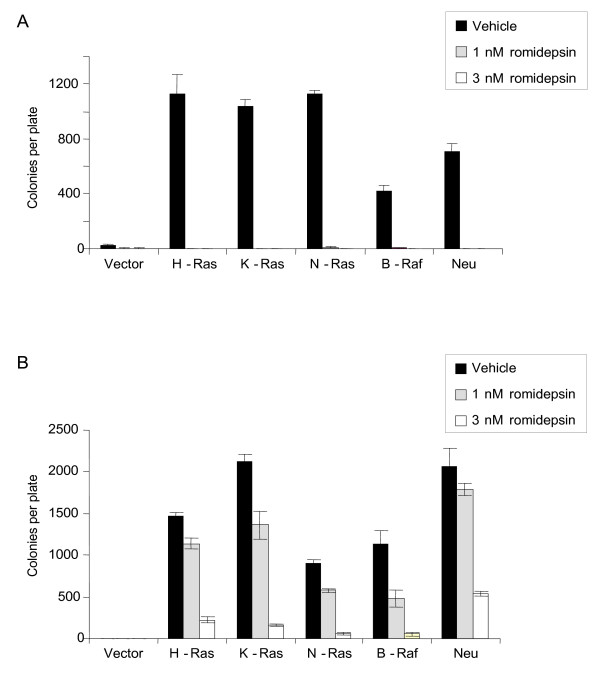
**Romidepsin inhibits anchorage-independent growth of transformed NIH 3T3 and RIE-1 cells**. NIH 3T3 cells (A) and RIE-1 cells (B) stably expressing the indicated proteins were evaluated for colony formation in soft agar in complete growth medium supplemented with the indicated concentrations of romidepsin. Following 23 d (NIH 3T3) or 15 d (RIE-1), viable colonies were stained in MTT and plates were scanned. The number of colonies was quantified from the scanned images using ImageJ software. Data shown are the average ± SD of triplicate plates and are representative of two independent experiments.

To extend previous biological studies, we analyzed the impact of romidepsin on the anchorage-independent growth of NIH 3T3 and RIE-1 cells. The ability of cells to survive and grow in soft agar, in an anchorage-independent manner, strongly correlates with tumorigenic growth potential *in vivo*. As expected, nontransformed (vector control) NIH 3T3 and RIE-1 cells displayed limited growth in the soft agar assay, whereas all oncogene-transformed cells exhibited efficient colony formation activity (Figure 4). For all transformed NIH 3T3 cell lines, 1 nM romidepsin effectively eliminated growth, reducing colony formation by over 95%. Therefore, romidepsin more potently inhibited the anchorage-independent growth of both Ras- and B-Raf-transformed NIH 3T3 cells than the anchorage-dependent growth of these cells.

For oncogene-transformed RIE-1 cells, 1 nM romidepsin only moderately reduced anchorage-independent growth (14-57% reduction in colonies; Figure 4B), in contrast to the strong reduction in soft agar growth of the transformed NIH 3T3 cells at this concentration. Treatment with 3 nM romidepsin dramatically reduced the anchorage-independent growth of all transformed RIE-1 cells (74-95% reduction in colonies; Figure 4B), consistent with the observations in NIH 3T3 cells, where romidepsin more potently inhibits anchorage-independent growth than anchorage-dependent growth.

### Romidepsin causes partial morphological reversion in RIE-1 cells

**Figure 5:**
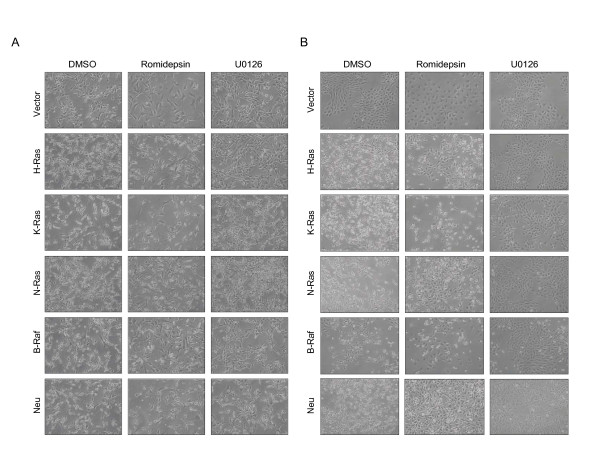
**Romidepsin more significantly alters the morphology of RIE-1 cells than that of NIH 3T3 cells**. NIH 3T3 (A) or RIE-1 (B) cells stably expressing the indicated proteins were incubated in complete growth medium supplemented with DMSO (Vehicle), 5 nM romidepsin, or 10 μM U0126 for 48 h. Representative fields of cells were photographed under 10× magnification. Data shown are representative of at least two independent experiments.

A hallmark of Ras transformation of NIH 3T3 and RIE-1 cells is the appearance of dramatic changes in cell morphology, reflecting reduced cell attachment and spreading. This morphology can be reverted to a more normal morphology with pharmacologic inhibitors of Ras signaling, for example, inhibitors of MEK activation of ERK [33,34]. To determine whether romidepsin can cause morphological reversion of cells transformed by K- and N-Ras, we treated our NIH 3T3 stable cell lines with 1, 5 or 10 nM romidepsin for 48 h (Figure 5 and data not shown). While romidepsin caused slight cell flattening and changes in cell shape (as determined by phase contrast microscopy), it did not cause substantial morphological reversion in NIH 3T3 cells. In contrast, treatment with the MEK inhibitor U0126, which blocks ERK activation, caused more substantial morphological reversion in the Ras- and Raf-transformed NIH-3T3 cells (Figure 5A), suggesting that romidepsin functions by a mechanism distinct from inhibition of Ras- or Raf-mediated MEK-ERK activation.

We also asked whether romidepsin could reverse the transformed morphology of RIE-1 cells. In contrast to the NIH 3T3 cells, 5 nM romidepsin did cause significant morphological changes in the RIE-1 cells (Figure 5B). Romidepsin treatment caused significant cell spreading in the nontransformed cells. Romidepsin altered the morphology of the cells transformed by the three different Ras isoforms to varying degrees: while romidepsin had minimal effects on the morphology of K-Ras-transformed cells, it caused morphological reversion (characterized by a decrease in cell refractility, and in increase in cell spreading, amount of cytoplasm, and cell size) in ~20% of H-Ras-transformed cells and caused substantial morphological reversion (greater than 50%) in the N-Ras- and ErbB2/Neu-transformed RIE-1 cells (Figure 5B). Similar results were obtained following 72 h treatment with romidepsin (data not shown). In contrast, treatment with U0126 caused near-complete morphological reversion of all transformed RIE-1 cells (Figure 5B). These data reveal isoform-specific differences in Ras-mediated transformation and show that romidepsin can partially revert the morphology of Ras-transformed RIE-1, but not NIH 3T3, cells.

### Romidepsin does not disrupt endogenous or ectopic expression of Ras or B-Raf

**Figure 6:**
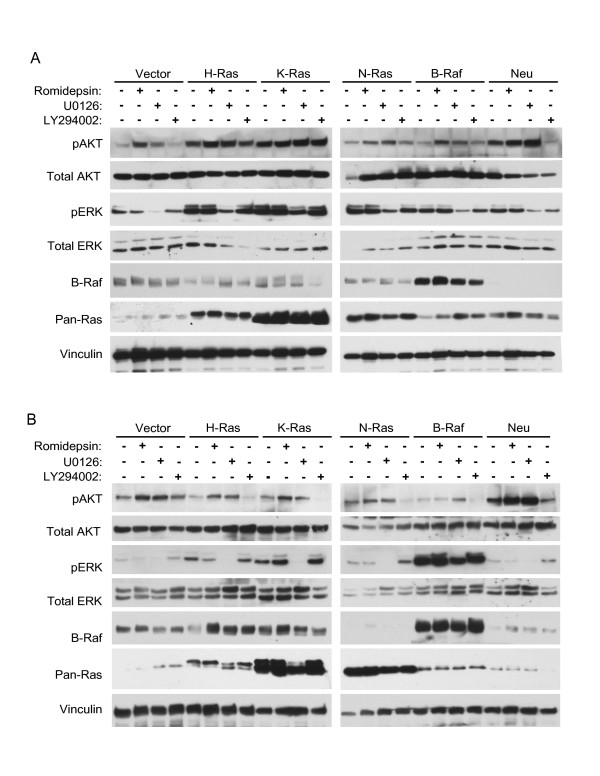
**Romidepsin does not inhibit Ras-mediated ERK or AKT activation in NIH 3T3 and RIE-1 cells**. NIH 3T3 (A) or RIE-1 (B) cells stably expressing the indicated proteins were treated with DMSO (Vehicle), 5 nM romidepsin, 10 μM U0126, or 10 μM LY294002 for 24 h. Cell lysates were analyzed by western blotting with antibodies to total ERK1/2, phospho-ERK1/2, total AKT, phospho-Akt, B-Raf, pan-Ras, and vinculin (loading control). Data shown are representative at least two independent experiments.

A previous study found that romidepsin treatment of human lung carcinoma cell lines reduced the steady-state levels of various signaling proteins, including Raf-1, which may account for inhibition of ERK signaling [21]. To determine whether romidepsin inhibition of Ras transformation involved loss of protein stability, we treated the NIH 3T3 and RIE-1 stable cell lines with 5 nM romidepsin for 24 h and probed lysates with antibodies to Ras and B-Raf. As expected, Ras levels were increased in the cells stably infected with retrovirus vectors encoding activated H-, K-, or N-Ras, with K-Ras levels being the highest (Figures 6A and 6B). Romidepsin treatment did not change the steady-state levels of endogenous or ectopic Ras protein. Similarly, B-Raf levels were increased in mutant B-Raf-transformed cells and were not changed with romidepsin treatment. These results confirmed continued expression of the endogenous and ectopically expressed oncoproteins and show that romidepsin treatment did not promote their degradation and loss.

### Romidepsin does not inhibit Ras signaling to ERK or AKT

Since previous studies suggested that romidepsin may impair Ras-mediated activation of these two pathways [17,21-23], we determined whether romidepsin treatment inhibited Ras-induced activation of ERK or AKT in our cells. We treated the transformed NIH 3T3 and RIE-1 cells with 5 nM romidepsin (to be consistent with previous studies [17]) for 24 h and probed lysates with antibodies to phospho-ERK and phospho-AKT. In the absence of romidepsin, phospho-ERK levels were increased in all transformed NIH 3T3 and RIE-1 cell lines compared to the nontransformed cells (Figure 6B). The MEK inhibitor U0126, a control for phospho-ERK inhibition, efficiently reduced levels of phospho-ERK in both NIH 3T3 and RIE-1 cells (Figures 6A and 6B). In contrast, romidepsin did not significantly change phospho-ERK levels. Thus, our results contrast with some previous studies and argue that inhibition of ERK activation does not contribute to the anti-proliferative activity of romidepsin.

As we described previously [35], Ras transformation caused increased AKT activation in NIH 3T3 but not RIE-1 cells (Figure 6). However, phospho-AKT levels were increased in ErbB2/Neu-transformed RIE-1 cells. Phospho-AKT levels were efficiently reduced by the PI3K inhibitor LY294002 in all transformed RIE-1 cells and some NIH 3T3 cells. Interestingly, both romidepsin and U0126 slightly increased the levels of phospho-AKT compared to DMSO-treated NIH 3T3 and RIE-1 cells. Taken together, our data suggest that romidepsin is not directly inhibiting Ras transformation through the Raf-MEK-ERK or PI3K-AKT pathway, and may instead disrupt transformation by other means.

## Discussion

Previous studies found that romidepsin displayed anti-Ras activities when evaluated in H-Ras-transformed mouse fibroblast model cell systems. However, whether this inhibition is mediated by inhibition of specific Ras effector signaling activities and whether this activity is associated with cell cycle inhibition or enhanced apoptosis is unclear. Furthermore, whether romidepsin can block transformation mediated by the Ras isoforms most commonly mutated in human cancers has not been addressed. To perform a rigorous evaluation of the anti-Ras activities of romidepsin, we studied the effects of romidepsin treatment on two well-established and characterized model cell systems that have been used extensively to study Ras signaling and the cellular consequences of signal transduction inhibitors. With growing evidence for Ras isoform differences in oncogenesis and well-established cell context differences in Ras-mediated oncogeneis, an important focus of our studies was the evaluation of K-Ras- and N-Ras-transformed NIH 3T3 fibroblasts and RIE-1 epithelial cells. Our results showed that romidepsin: 1) blocks transformation induced by H-Ras as well as K-Ras and N-Ras, 2) also blocks growth transformation induced by B-Raf and ErbB2/Neu, 3) does not block canonical Ras effector signaling pathways, and 4) impedes growth transformation by promoting cell cycle arrest and not apoptosis.

We utilized two well-characterized model cell systems of oncogenic Ras signaling and transformation. NIH 3T3 cells have been used extensively as a model system to study Ras transformation [36,37]. Furthermore, some of the early studies on the effects of romidepsin on Ras transformation were performed in this cell line [16]. Ras transformation has also been studied in the RIE-1 epithelial cell line [37-39], and a similar intestinal epithelial cell line (IEC) was used recently to study the effects of other HDAC inhibitors on Ras transformation [40]. The Ras effector pathway critical for growth transformation of these cell lines has also been established, and a key role for the Raf effector pathway has been established in both cell types [20]. In light of cell context differences in Ras function [41], our studies provide a comparison of romidepsin activity in the fibroblastic cell type most widely used in previous studies with activities seen in an epithelial cell line, the cell type from which the majority of *RAS *mutation-positive cancers arise. The advantages of using such model cell systems include the fact that mutationally-activated Ras alone is sufficient to cause full one-step growth transformation. Thus, while such systems lack the genetic complexity of authentic tumor cells, these model systems have the great advantage in possessing a clear role for mutated Ras signaling in the continued maintenance of growth transformation.

We showed that romidepsin inhibited both the anchorage-dependent and anchorage-independent growth of NIH 3T3 and RIE-1 cells transformed by all three Ras isoforms, as well as by B-Raf and ErbB2/Neu. Since B-Raf is a downstream effector of Ras, B-Raf-induced transformation is not expected to depend on Ras. Therefore, these results suggest that romidepsin is not a specific Ras inhibitor. In NIH 3T3 mouse fibroblasts, we observed that romidepsin more potently inhibited the anchorage-dependent proliferation of transformed cells when compared to nontransformed cells. In contrast, in the RIE-1 epithelial cell line, we observed the same degree of anchorage-dependent growth inhibition in nontransformed, B-Raf-transformed, and Ras-transformed cells. These results are not entirely surprising, given that differences in Ras-induced transformation between NIH 3T3 and RIE-1 cells have been well documented [25,33]. The striking differences we observed in NIH 3T3 fibroblasts versus RIE-1 epithelial cells highlight the value of using two model cell systems to study inhibition of transformation and suggest that caution should be used when extrapolating information gleaned from a single cell line to other systems. Our results also suggest that while romidepsin does block Ras-mediated growth transformation, it also blocks transformation induced by other oncogenes, and thus we do not expect that patient response to romidepsin will be limited to tumors harboring *RAS *mutations.

Recently, Klampfer *et al. *reported that two other HDAC inhibitors, butyrate and suberoylanilide hydroxamic acid (SAHA), selectively induced apoptosis under anchorage-dependent conditions in IEC-6 rat intestinal epithelial cells transformed with K-Ras, compared to nontransformed cells [40]. These data contrast with our results in RIE-1 cells where we found that nontransformed cells were as sensitive to romidepsin as Ras-transformed cells. This discrepancy may be due to differences in the properties of inhibitors used (SAHA and romidepsin are not equivalent, and have entirely different chemical structures), the cell lines studied, and the assays used to monitor apoptosis (Klampfer *et al. *used flow cytometry to measure apoptosis, whereas we used caspase-3 and PARP cleavage to detect apoptosis). Other groups have also reported that romidepsin selectively induces caspase-3 cleavage, PARP cleavage, and apoptosis in Ras-transformed cells [10,17,24,28]. However, we failed to observe cleavage of either caspase-3 or PARP in response to romidepsin in either vector control or Ras-transformed cells under the conditions in which romidepsin blocked cell proliferation. In previous studies, 10T1/2 and J82 cell lines were used, whereas we studied NIH 3T3 and RIE-1 cell lines, so the differences in cell lines may account for the differences in results. Indeed, in our studies here we have shown that romidepsin treatment can have strikingly different effects in NIH 3T3 cells compared to RIE-1 cells. It remains possible that higher concentrations of romidepsin or different tissue culture conditions (such as serum-starvation) may induce apoptosis in these cells, but we conclude that apoptosis did not significantly contribute to the decrease in anchorage-dependent or -independent growth that we observed with romidepsin. Instead, we observed that romidepsin increased levels of the CDK inhibitor p21 and decreased expression of cyclin D1, suggesting that romidepsin blocks cell proliferation by inhibiting cell cycle progression rather than by induction of apoptosis. Furthermore, we found that romidepsin treatment decreased phosphorylation of Rb, which correlates with arrest in the G_1 _phase of the cell cycle. These observations are consistent with observations from clinical trial analyses, where HDAC inhibitors may cause growth arrest in some settings and apoptosis in others. In summary, our results suggest that *RAS *mutation status alone will not be a reliable genetic marker to predict tumor cell response to HDAC inhibition.

Previous studies have shown that romidepsin reverts the morphology of H-Ras-transformed NIH 3T3 mouse fibroblasts [16]. While we did observe some morphological changes, we did not observe substantial morphological reversion to the nontransformed phenotype in Ras-transformed NIH 3T3 cells treated with romidepsin. In contrast, treatment with the U0126 MEK inhibitor did result in substantial morphological reversion, providing further evidence against an involvement of ERK inhibition to romidepsin activity. Differences in experimental conditions and in strain differences in the NIH 3T3 cells utilized [42] may account for these discrepancies with our NIH 3T3 results. For example, Ueda *et al. *transformed cells using transfection of genomic DNA from human bladder carcinoma EJ cells (which contain a mutated *HRAS *allele) [16], whereas we retrovirally infected cells with H-, K-, or N-Ras cDNA to obtain Ras-transformed cells. These differences in Ras transformation may contribute to the differences in results. In contrast, we did observe partial morphological reversion in transformed RIE-1 cells, particularly those transformed with ErbB2/Neu and with N-Ras. The greater sensitivity of N-Ras-transformed RIE-1 intestinal epithelial cells to reversion may reflect Ras isoform functional differences, for example, as described for K-Ras and N-Ras in oncogenesis in a mouse model for colon cancer development [19].

While several previous studies have investigated the effects of romidepsin on Ras activation of the Raf and PI3K effector pathways, as measured by phosphorylation of ERK and AKT, no consensus has been reached. In some studies, romidepsin has been reported to decrease levels of phospho-ERK in H-Ras-transformed cells and in cancer cell lines [17,21,23], whereas in others, no alterations in the levels of phospho-ERK in Ras-transformed cells were seen [24]. In nontransformed cells, romidepsin was shown to increase phospho-ERK [17,24]. We did not observe significant changes in phospho-ERK in both the RIE-1 and NIH 3T3 transformed and nontransformed cells. Though some reported differences may be due to differences in romidepsin dosing regimens, we tested three growth-inhibitory concentrations of romidepsin (2, 5, and 10 nM) at three different time periods (6, 24, and 48 h), and did not observe any differences in phospho-ERK (data not shown). We also observed that romidepsin slightly increased levels of phospho-AKT in most cell lines tested, in agreement with Song *et al *[24]. This contrasts with data from Fecteau *et al*., who reported that romidepsin increased phospho-Akt in nontransformed cells, but decreased phospho-AKT in Ras-transformed C3H10T1/2 cells [17]. Again, the discrepancies may be due to differences in cell type or cell strain, concentration of drug, or length of treatment. For example, Fecteau *et al. *used 10T1/2 cells, which in our own analyses do exhibit different properties than NIH 3T3 cells. Yu *et al. *and Kobayashi *et al. *both used much higher concentrations of romidepsin to elicit a decrease in ERK phosphorylation (25 ng/mL, which is equivalent to 46 nM, and 100 nM, respectively) [21,23]. Indeed, Kobayashi *et al. *did not observe a decrease in ERK phosphorylation using just 10 nM of romidepsin, consistent with our studies where 5 nM did not reduce ERK activity, yet did reduce growth. Regardless, our results demonstrate that in our cell models, romidepsin is not inhibiting growth and transformation by directly inhibiting these two key Ras effector signaling pathways. Thus, we suggest that ERK and AKT phosphorylation will not be useful biomarkers for romidepsin activity. Since at least three additional Ras effector pathways have been implicated in oncogenesis (e.g., the RalGEF-Ral pathway), we cannot exclude the possibility that romidepsin may block other Ras effector pathways. However, these effectors do not potently transform NIH 3T3 or RIE-1 cells, making it unlikely that romidepsin inhibition of Ras involves antagonism of Ras effector activation.

We did not find evidence for romidepsin inhibition of Ras signaling associated with inhibition of Ras transformation. Thus, the mechanism for romidepsin inhibition of Ras-induced transformation in our models remains unclear. Neoplastic transformation depends on activation of oncogenes as well as on silencing of tumor suppressor genes [43]. Ras-induced transformation requires epigenetic events that direct silencing of tumor suppressor and pro-apoptotic genes [44]. Recently, Ras has been shown to silence gene expression using a pathway consisting of many chromatin modifiers, including HDAC9 [44]. HDAC inhibitors such as romidepsin are known to reverse the epigenetic changes that have occurred in tumor cells, restoring them to a more normal state. Although romidepsin displays poor activity against the HDAC9 isoform, other HDAC isoforms may be required for Ras-induced transformation. For example, HDAC6 was recently found to be required for Ras-induced transformation [45]. HDAC inhibitors are also known to increase expression of several cell cycle inhibitors, including p21^CIP1/WAF1 ^[6]. These changes in gene expression may account for the potent growth inhibition of Ras- and Raf-transformed cells that we observed with romidepsin. Future gene array analyses to assess the gene expression changes seen in romidepsin-treated nontransformed and Ras-transformed NIH 3T3 and RIE-1 cells will be important to further evaluate this issue.

## Conclusion

In summary, while our studies showed romidepsin activities that differed from observations made by other studies, our main conclusion, that Ras-transformed cells are sensitive to romidepsin treatment, is in agreement with previous studies. Our distinct observations made with Ras-transformed NIH 3T3 and RIE-1 cells demonstrate the striking cell context dependence of romidepsin action. This may also account for the highly varied observations made in the literature concerning the multiple mechanisms of anti-Ras activities described for romidepsin and highlights the importance of using more than one model system. We also observed that romidepsin blocked transformation induced by B-Raf, suggesting that the anti-tumor activity of romidepsin is not specific for transformation driven by Ras. Therefore, we suggest that *RAS *mutation status alone will not be a reliable molecular determinant to predict sensitivity to romidepsin. Further analysis of romidepsin anti-tumor activity in human tumor cell lines with and without *RAS *mutations will be required to confirm this prediction.

## Abbreviations

CDK: cyclin-dependent kinase; DMEM: Dulbecco's modified Eagle's medium; DMSO: dimethyl sulfoxide; EDTA: ethylenediaminetetraacetic acid; ERK: extracellular signal-regulated kinase; GDP: guanosine diphosphate; GEF: guanine nucleotide exchange factor; GTP: guanosine triphosphate; HDAC: histone deacetylase; MTT: 3-(4,5-dimethyl-2-thiazolyl)-2,5-diphenyl-2H-tetrazolium bromide; PARP: poly (ADP-ribose) polymerase; PBS: phosphate buffered saline; PI3K: phosphatidylinositol 3-kinase; PIP_2_: phosphatidylinositol (4,5) bisphosphate; PIP_3_: phosphatidylinositol (3,4,5) triphosphate; RIE: rat intestinal epithelial; Rb: retinoblastoma; SAHA: suberoylanilide hydroxamic acid; SDS-PAGE: sodium dodecyl sulfate-polyacrylamide gel electrophoresis; STS: staurosporine.

## Competing interests

C.J.D. has received an unrestricted research contribution (less than $10,000) from Gloucester Pharmaceuticals.

## Authors' contributions

ABH carried out all cellular and biochemical studies and drafted the manuscript. KDH generated the stable cell lines, performed the initial cellular growth inhibition assays with NIH 3T3 and RIE-1 cells, and established the general direction of the studies. JN and CJD conceived of the study and participated in its design, and CJD coordinated the study and assisted in the drafting of the manuscript. All authors read and approved the final manuscript.
